# (*R*)-1,1′-Binaphthalene-2,2′-diyl dicinnamate

**DOI:** 10.1107/S1600536808008878

**Published:** 2008-04-10

**Authors:** Wen Weng, Hong-Xu Guo, Zhi-Fen Chen, Qing-Hua Wang, Bi-Xia Yao

**Affiliations:** a Department of Chemistry and Environmental Science, Zhangzhou Normal University, Zhangzhou, Fujian 363000, People’s Republic of China

## Abstract

In the title compound, C_38_H_26_O_4_, two cinnamo­yloxy groups are linked in a *trans* fashion to the two O atoms of optically active (*R*)-1,10-bi-2-naphthol. The dihedral angle between the mean planes of the two naphthyl groups is 71.8 (1)°. The crystal structure contains inter­molecular C—H⋯O and C—H⋯π inter­actions.

## Related literature

For related literature, see: Chu *et al.* (2001 ▶); Goldberg (1980 ▶); Horikoshi *et al.* (2004 ▶); Lee & Lin (2002 ▶); Luo *et al.* (2002 ▶); Noyori (2002 ▶); Pu (1998 ▶).
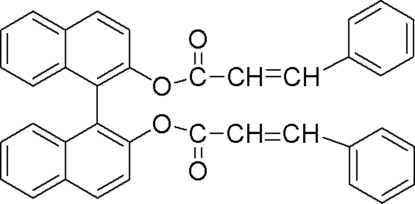


## Experimental

### 

#### Crystal data

C_38_H_26_O_4_
*M* *_r_* = 546.59
Orthorhombic, 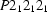

*a* = 10.3391 (17) Å
*b* = 15.352 (2) Å
*c* = 17.660 (3) Å
*V* = 2803.1 (8) Å^3^
*Z* = 4
Mo *K*α radiation
μ = 0.08 mm^−1^
*T* = 293 (2) K
0.52 × 0.43 × 0.38 mm

#### Data collection

Siemens SMART CCD diffractometer
Absorption correction: multi-scan (*SADABS*; Sheldrick, 1996 ▶) *T*_min_ = 0.936, *T*_max_ = 0.969
27455 measured reflections
2894 independent reflections
2753 reflections with *I* > 2σ(*I*)
*R*_int_ = 0.037

#### Refinement

*R*[*F* ^2^ > 2σ(*F* ^2^)] = 0.043
*wR*(*F* ^2^) = 0.143
*S* = 1.02
2894 reflections
381 parameters
H-atom parameters constrained
Δρ_max_ = 0.14 e Å^−3^
Δρ_min_ = −0.15 e Å^−3^

### 

Data collection: *SMART* (Siemens, 1994 ▶); cell refinement: *SAINT* (Siemens, 1994 ▶); data reduction: *SAINT*; program(s) used to solve structure: *SHELXS97* (Sheldrick, 2008 ▶); program(s) used to refine structure: *SHELXL97* (Sheldrick, 2008 ▶); molecular graphics: *SHELXL97*; software used to prepare material for publication: *SHELXL97*.

## Supplementary Material

Crystal structure: contains datablocks I, global. DOI: 10.1107/S1600536808008878/bi2285sup1.cif

Structure factors: contains datablocks I. DOI: 10.1107/S1600536808008878/bi2285Isup2.hkl

Additional supplementary materials:  crystallographic information; 3D view; checkCIF report

## Figures and Tables

**Table 1:** Hydrogen-bond geometry (Å, °)

*D*—H⋯*A*	*D*—H	H⋯*A*	*D*⋯*A*	*D*—H⋯*A*
C23—H23*A*⋯O1	0.93	2.38	2.736 (3)	103
C32—H32*A*⋯O4	0.93	2.49	2.833 (4)	102
C11—C11A⋯*Cg*1^i^	0.93	2.85	3.746 (3)	162
C2—H2*A*⋯*Cg*2^ii^	0.93	2.74	3.507 (3)	140

Symmetry codes: (i) 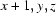; (ii) 
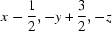
.

**Fig. 1.**
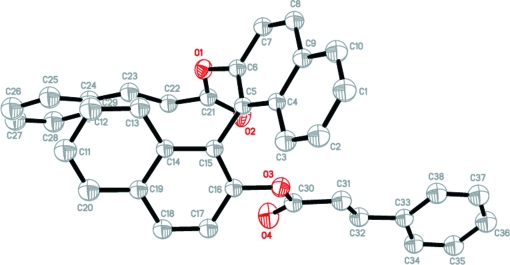
The molecular structure with displacement ellipsoids drawn at the 50% probability level for non-H atoms.

**Fig. 2.**
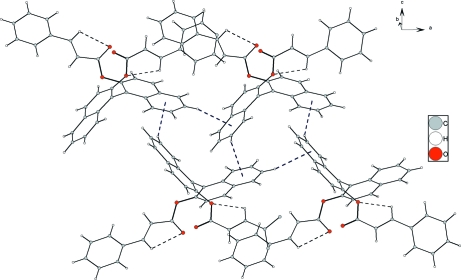
View of the hydrogne-bond packing for compound (I), showing C—H···O and C—H···π contact between molecules as dashed lines.

## supplementary crystallographic information

### Comment

Optically active 1,10-bi-2-naphthol (BINOL) derivatives have been used successfully in asymmetric catalysis, molecular recognition and optical materials (Pu, 1998; Chu *et al.*, 2001; Luo *et al.*, 2002; Lee & Lin, 2002; Noyori, 2002). Their success is due to the fact that the axial chirality of the ligands can be well expressed in the steric environment of the active sites, and the chiral configuration of BINOL molecules is known to be stable at high temperature over extended periods of time. Thus, BINOL may be used as a preferred starting material or auxiliary for the synthesis of homochiral functional supramolecular complexes (Horikoshi *et al.*, 2004). Here we report the synthesis and crystal structure of the homochiral title compound.

The compound is composed of two cinnamoyloxy units linked in a *trans* fashion to the two O atoms (2,2'-) of the optically active (*R*)-BINOL (Fig. 1). The bond distances C6—O1 and C16—O3 are 1.399 (3) and 1.403 (4) Å, respectively. The separation between atoms O1 and O3 is 4.230 (3) Å, which is longer than that reported in other 2,2'-O-substituted complexes (Goldberg, 1980). Considerable twisting between the two naphthyl groups in the compound produces a dihedral angle 71.8 (1)°, much less than the angle of 101.7° found in (*R*)-BINOL itself. The naphthyl groups are also highly twisted with respect to their covalently linked phenyl groups, with dihedral angles of 28.6 (1) and 74.2 (1)°, respectively. These twists may be ascribed to steric repulsion, resulting in the two cinnamoyloxy units lying on opposite sides of the binaphthyl backbone.

The crystal structure contains C—H···O and C—H···π interactions (Fig. 2 and Table 1). Denoting the centroids of rings [C1–C4, C9, C10], [C11–C14, C19, C20], [C24–C29] and [C33–C38] as *Cg*1, *Cg*2, C*g*3 and C*g*4, respectively, the centroid-centroid distances are: *Cg*2···*Cg*1^i^ = 4.749 (2) Å, *Cg*3···*Cg*4^ii^ = 4.716 (2) Å [symmetry codes (i): 1/2 + *x*, 3/2 - *y*, -*z*; (ii) 1 + *x*, *y*, *z*].

### Experimental

To a 50 ml round-bottom flask was added 2.0 g (7.0 mmol) of (*R*)-1,1'-bi-2-naphthol, 20 ml THF and 6.6 ml pyridine. Then, 5.0 ml *trans*-cinnamoyl chloride (25.9 mmol) was added in an ice bath. The mixture was stirred at ambient temperature for 24 h, and then poured onto ice. The resulting solid was filtered and washed with hot water. The crude product was soaked with absolute methanol twice to afford the target compound, which was recrystallized from THF/MeOH to afford colourless blocks.

### Refinement

H atoms were positioned geometrically (C—H = 0.93 Å) and allowed to ride on their respective parent C atoms with *U*_iso_(H) = 1.2 *U*_eq_(C). In the absence of significant anomalous scattering effects, 2231 Friedel pairs have been merged.

### Crystal data

**Table:**

C_38_H_26_O_4_	*F*_000_ = 1144
*M**_r_* = 546.59	*D*_x_ = 1.295 Mg m^−^^3^
Orthorhombic, *P*2_1_2_1_2_1_	Mo *K*α radiation λ = 0.71070 Å
Hall symbol: P 2ac 2ab	Cell parameters from 27455 reflections
*a* = 10.3391 (17) Å	θ = 3.0–25.4º
*b* = 15.352 (2) Å	µ = 0.08 mm^−^^1^
*c* = 17.660 (3) Å	*T* = 293 (2) K
*V* = 2803.1 (8) Å^3^	Block, colourless
*Z* = 4	0.52 × 0.43 × 0.38 mm

### Data collection

**Table:**

Siemens SMART CCD diffractometer	2894 independent reflections
Radiation source: fine-focus sealed tube	2753 reflections with *I* > 2σ(*I*)
Monochromator: graphite	*R*_int_ = 0.037
*T* = 293(2) K	θ_max_ = 25.4º
ω scans	θ_min_ = 3.0º
Absorption correction: multi-scan(SADABS; Sheldrick, 1996)	*h* = −12→12
*T*_min_ = 0.936, *T*_max_ = 0.969	*k* = −18→18
27455 measured reflections	*l* = −18→21

### Refinement

**Table:**

Refinement on *F*^2^	Hydrogen site location: inferred from neighbouring sites
Least-squares matrix: full	H-atom parameters constrained
*R*[*F*^2^ > 2σ(*F*^2^)] = 0.043	*w* = 1/[σ^2^(*F*_o_^2^) + (0.12*P*)^2^] where *P* = (*F*_o_^2^ + 2*F*_c_^2^)/3
*wR*(*F*^2^) = 0.144	(Δ/σ)_max_ < 0.001
*S* = 1.02	Δρ_max_ = 0.14 e Å^−^^3^
2894 reflections	Δρ_min_ = −0.15 e Å^−^^3^
381 parameters	Extinction correction: SHELXL97 (Sheldrick, 1997), Fc^*^=kFc[1+0.001xFc^2^λ^3^/sin(2θ)]^-1/4^
Primary atom site location: structure-invariant direct methods	Extinction coefficient: 0.013 (3)
Secondary atom site location: difference Fourier map	

### Special details

**Table:**

Geometry. All e.s.d.'s (except the e.s.d. in the dihedral angle between two l.s. planes) are estimated using the full covariance matrix. The cell e.s.d.'s are taken into account individually in the estimation of e.s.d.'s in distances, angles and torsion angles; correlations between e.s.d.'s in cell parameters are only used when they are defined by crystal symmetry. An approximate (isotropic) treatment of cell e.s.d.'s is used for estimating e.s.d.'s involving l.s. planes.
Refinement. Refinement of *F*^2^ against ALL reflections. The weighted *R*-factor *wR* and goodness of fit *S* are based on *F*^2^, conventional *R*-factors *R* are based on *F*, with *F* set to zero for negative *F*^2^. The threshold expression of *F*^2^ > σ(*F*^2^) is used only for calculating *R*-factors(gt) *etc*. and is not relevant to the choice of reflections for refinement. *R*-factors based on *F*^2^ are statistically about twice as large as those based on *F*, and *R*- factors based on ALL data will be even larger.

### Fractional atomic coordinates and isotropic or equivalent isotropic displacement parameters (Å^2^)

**Table:**

	*x*	*y*	*z*	*U*_iso_*/*U*_eq_	
C1	0.6025 (3)	0.8638 (3)	−0.04126 (18)	0.0563 (8)	
H1A	0.5442	0.8445	−0.0777	0.068*	
C2	0.6764 (3)	0.8040 (2)	−0.00129 (17)	0.0535 (8)	
H2A	0.6671	0.7448	−0.0114	0.064*	
C3	0.7621 (3)	0.8302 (2)	0.05227 (17)	0.0477 (7)	
H3A	0.8100	0.7889	0.0787	0.057*	
C4	0.7794 (3)	0.91986 (18)	0.06838 (15)	0.0380 (6)	
C5	0.8758 (3)	0.95009 (17)	0.12052 (14)	0.0373 (6)	
C6	0.8883 (3)	1.03778 (19)	0.13057 (15)	0.0394 (6)	
C7	0.8090 (3)	1.09864 (19)	0.09384 (16)	0.0450 (7)	
H7A	0.8187	1.1578	0.1037	0.054*	
C8	0.7186 (3)	1.0711 (2)	0.04412 (17)	0.0454 (7)	
H8A	0.6661	1.1116	0.0198	0.055*	
C9	0.7027 (3)	0.9807 (2)	0.02847 (15)	0.0421 (6)	
C10	0.6151 (3)	0.9511 (2)	−0.02713 (17)	0.0511 (8)	
H10A	0.5658	0.9910	−0.0543	0.061*	
C11	1.3620 (3)	0.8723 (3)	0.1022 (2)	0.0603 (9)	
H11A	1.4498	0.8707	0.0910	0.072*	
C12	1.2803 (3)	0.9291 (2)	0.0633 (2)	0.0586 (9)	
H12A	1.3144	0.9643	0.0254	0.070*	
C13	1.1519 (3)	0.9341 (2)	0.07945 (18)	0.0477 (7)	
H13A	1.0997	0.9725	0.0525	0.057*	
C14	1.0972 (3)	0.88157 (17)	0.13694 (15)	0.0386 (6)	
C15	0.9642 (3)	0.88757 (17)	0.15923 (15)	0.0371 (6)	
C16	0.9218 (3)	0.83490 (18)	0.21667 (16)	0.0399 (6)	
C17	1.0014 (3)	0.77282 (19)	0.25182 (18)	0.0469 (7)	
H17A	0.9679	0.7362	0.2889	0.056*	
C18	1.1267 (3)	0.7668 (2)	0.23135 (17)	0.0493 (7)	
H18A	1.1791	0.7255	0.2547	0.059*	
C19	1.1804 (3)	0.82197 (18)	0.17505 (17)	0.0431 (6)	
C20	1.3128 (3)	0.8194 (2)	0.15641 (19)	0.0537 (8)	
H20A	1.3672	0.7809	0.1817	0.064*	
C21	0.9902 (3)	1.05583 (19)	0.24984 (17)	0.0431 (7)	
C22	1.1166 (3)	1.0709 (2)	0.28411 (18)	0.0475 (7)	
H22A	1.1209	1.0753	0.3366	0.057*	
C23	1.2250 (3)	1.0785 (2)	0.24563 (18)	0.0498 (8)	
H23A	1.2183	1.0785	0.1931	0.060*	
C24	1.3556 (3)	1.0870 (2)	0.27757 (19)	0.0518 (8)	
C25	1.4610 (3)	1.0705 (3)	0.2321 (2)	0.0665 (10)	
H25A	1.4485	1.0593	0.1809	0.080*	
C26	1.5852 (4)	1.0704 (3)	0.2613 (3)	0.0771 (12)	
H26A	1.6550	1.0573	0.2301	0.093*	
C27	1.6053 (4)	1.0894 (3)	0.3354 (3)	0.0697 (10)	
H27A	1.6888	1.0894	0.3549	0.084*	
C28	1.5032 (4)	1.1086 (3)	0.3813 (2)	0.0729 (11)	
H28A	1.5174	1.1222	0.4319	0.087*	
C29	1.3775 (3)	1.1078 (3)	0.3526 (2)	0.0654 (10)	
H29A	1.3083	1.1214	0.3841	0.078*	
C30	0.7596 (3)	0.8456 (2)	0.31233 (16)	0.0476 (7)	
C31	0.6190 (3)	0.8482 (2)	0.3223 (2)	0.0542 (8)	
H31A	0.5650	0.8516	0.2803	0.065*	
C32	0.5688 (3)	0.8457 (2)	0.3910 (2)	0.0526 (8)	
H32A	0.6282	0.8421	0.4305	0.063*	
C33	0.4326 (3)	0.8477 (2)	0.41384 (18)	0.0499 (7)	
C34	0.4012 (3)	0.8251 (2)	0.4876 (2)	0.0591 (8)	
H34A	0.4665	0.8079	0.5205	0.071*	
C35	0.2755 (4)	0.8276 (3)	0.5130 (2)	0.0668 (10)	
H35A	0.2562	0.8129	0.5628	0.080*	
C36	0.1783 (4)	0.8520 (2)	0.4639 (3)	0.0681 (10)	
H36A	0.0929	0.8528	0.4804	0.082*	
C37	0.2072 (4)	0.8749 (3)	0.3909 (2)	0.0661 (10)	
H37A	0.1413	0.8917	0.3581	0.079*	
C38	0.3337 (4)	0.8733 (2)	0.3654 (2)	0.0616 (9)	
H38A	0.3526	0.8893	0.3159	0.074*	
O1	0.99096 (19)	1.07037 (12)	0.17371 (12)	0.0440 (5)	
O2	0.8952 (2)	1.03255 (17)	0.28368 (13)	0.0579 (6)	
O3	0.79103 (19)	0.84135 (14)	0.23723 (11)	0.0462 (5)	
O4	0.8384 (2)	0.8467 (2)	0.36224 (13)	0.0664 (7)	

### Atomic displacement parameters (Å^2^)

**Table:**

	*U*^11^	*U*^22^	*U*^33^	*U*^12^	*U*^13^	*U*^23^
C1	0.0468 (16)	0.081 (2)	0.0407 (15)	−0.0084 (17)	−0.0064 (14)	−0.0052 (15)
C2	0.0578 (18)	0.0568 (18)	0.0458 (16)	−0.0116 (15)	−0.0009 (15)	−0.0114 (14)
C3	0.0511 (16)	0.0498 (16)	0.0424 (15)	−0.0003 (14)	−0.0014 (14)	−0.0005 (13)
C4	0.0367 (14)	0.0461 (14)	0.0313 (12)	−0.0033 (11)	0.0013 (11)	0.0017 (11)
C5	0.0392 (14)	0.0432 (14)	0.0294 (12)	−0.0002 (12)	0.0014 (11)	0.0024 (11)
C6	0.0373 (14)	0.0422 (14)	0.0388 (14)	0.0004 (12)	−0.0023 (12)	0.0007 (11)
C7	0.0480 (16)	0.0396 (14)	0.0474 (15)	0.0043 (12)	0.0003 (14)	0.0030 (12)
C8	0.0405 (14)	0.0503 (16)	0.0455 (15)	0.0072 (13)	−0.0025 (13)	0.0047 (12)
C9	0.0362 (13)	0.0574 (17)	0.0326 (13)	−0.0003 (12)	0.0034 (11)	0.0021 (12)
C10	0.0425 (16)	0.069 (2)	0.0416 (16)	0.0001 (15)	−0.0030 (13)	0.0034 (14)
C11	0.0436 (17)	0.074 (2)	0.063 (2)	0.0080 (16)	0.0087 (16)	−0.0057 (18)
C12	0.0595 (19)	0.0611 (19)	0.0552 (18)	−0.0013 (16)	0.0182 (16)	0.0022 (16)
C13	0.0498 (17)	0.0451 (15)	0.0483 (16)	0.0018 (13)	0.0078 (14)	0.0020 (13)
C14	0.0427 (14)	0.0372 (13)	0.0359 (13)	0.0021 (11)	−0.0020 (12)	−0.0041 (11)
C15	0.0410 (14)	0.0359 (12)	0.0344 (13)	−0.0011 (11)	−0.0034 (11)	−0.0010 (11)
C16	0.0394 (14)	0.0413 (14)	0.0389 (14)	0.0002 (11)	−0.0003 (12)	−0.0007 (12)
C17	0.0532 (17)	0.0441 (15)	0.0432 (15)	−0.0021 (14)	−0.0017 (13)	0.0067 (12)
C18	0.0564 (18)	0.0435 (15)	0.0480 (16)	0.0107 (14)	−0.0074 (15)	0.0074 (13)
C19	0.0436 (15)	0.0426 (14)	0.0432 (14)	0.0049 (12)	−0.0017 (13)	−0.0073 (12)
C20	0.0480 (16)	0.0589 (17)	0.0542 (18)	0.0118 (15)	0.0002 (15)	−0.0009 (15)
C21	0.0423 (15)	0.0469 (15)	0.0401 (15)	0.0055 (13)	−0.0008 (12)	−0.0054 (12)
C22	0.0436 (16)	0.0573 (18)	0.0415 (15)	0.0022 (13)	−0.0046 (13)	−0.0061 (14)
C23	0.0408 (16)	0.0619 (19)	0.0466 (16)	0.0031 (14)	−0.0057 (13)	−0.0020 (14)
C24	0.0412 (16)	0.0604 (18)	0.0536 (18)	−0.0027 (14)	−0.0008 (14)	0.0010 (15)
C25	0.0461 (18)	0.092 (3)	0.061 (2)	−0.0067 (18)	−0.0005 (16)	−0.007 (2)
C26	0.0386 (17)	0.107 (3)	0.086 (3)	−0.0026 (19)	0.0075 (18)	−0.011 (3)
C27	0.0407 (17)	0.085 (3)	0.084 (3)	−0.0079 (17)	−0.0131 (18)	0.003 (2)
C28	0.054 (2)	0.100 (3)	0.065 (2)	−0.007 (2)	−0.0151 (18)	−0.010 (2)
C29	0.0471 (17)	0.087 (3)	0.062 (2)	−0.0015 (18)	−0.0020 (16)	−0.0150 (19)
C30	0.0485 (16)	0.0566 (17)	0.0376 (14)	−0.0043 (14)	0.0049 (14)	0.0043 (13)
C31	0.0452 (16)	0.069 (2)	0.0489 (17)	−0.0060 (15)	0.0009 (14)	0.0040 (15)
C32	0.0481 (16)	0.0616 (18)	0.0481 (17)	0.0003 (15)	0.0016 (14)	0.0032 (15)
C33	0.0492 (16)	0.0509 (16)	0.0495 (17)	−0.0027 (14)	0.0012 (14)	−0.0018 (14)
C34	0.0522 (18)	0.070 (2)	0.0548 (19)	0.0006 (17)	0.0037 (16)	0.0054 (16)
C35	0.060 (2)	0.070 (2)	0.070 (2)	−0.0032 (18)	0.0194 (19)	0.0031 (18)
C36	0.054 (2)	0.059 (2)	0.091 (3)	−0.0054 (17)	0.018 (2)	−0.0091 (19)
C37	0.057 (2)	0.065 (2)	0.077 (2)	0.0075 (18)	−0.0123 (19)	−0.012 (2)
C38	0.064 (2)	0.065 (2)	0.0564 (19)	0.0058 (17)	−0.0020 (17)	−0.0009 (16)
O1	0.0415 (10)	0.0474 (10)	0.0432 (10)	−0.0053 (9)	−0.0061 (9)	−0.0015 (9)
O2	0.0426 (12)	0.0834 (16)	0.0478 (12)	−0.0039 (11)	0.0047 (10)	−0.0116 (11)
O3	0.0438 (11)	0.0553 (11)	0.0395 (10)	−0.0036 (9)	−0.0010 (9)	0.0037 (9)
O4	0.0557 (13)	0.102 (2)	0.0418 (12)	0.0007 (13)	−0.0019 (11)	−0.0032 (12)

### Geometric parameters (Å, °)

**Table:**

C1—C10	1.371 (5)	C21—O2	1.204 (4)
C1—C2	1.388 (5)	C21—O1	1.363 (4)
C1—H1A	0.930	C21—C22	1.459 (4)
C2—C3	1.357 (4)	C22—C23	1.316 (4)
C2—H2A	0.930	C22—H22A	0.930
C3—C4	1.417 (4)	C23—C24	1.469 (4)
C3—H3A	0.930	C23—H23A	0.930
C4—C9	1.414 (4)	C24—C25	1.376 (5)
C4—C5	1.434 (4)	C24—C29	1.382 (5)
C5—C6	1.364 (4)	C25—C26	1.384 (5)
C5—C15	1.491 (4)	C25—H25A	0.930
C6—O1	1.399 (3)	C26—C27	1.355 (6)
C6—C7	1.402 (4)	C26—H26A	0.930
C7—C8	1.350 (4)	C27—C28	1.363 (6)
C7—H7A	0.930	C27—H27A	0.930
C8—C9	1.425 (4)	C28—C29	1.395 (5)
C8—H8A	0.930	C28—H28A	0.930
C9—C10	1.411 (4)	C29—H29A	0.930
C10—H10A	0.930	C30—O4	1.201 (4)
C11—C20	1.354 (5)	C30—O3	1.367 (3)
C11—C12	1.396 (5)	C30—C31	1.465 (5)
C11—H11A	0.930	C31—C32	1.320 (5)
C12—C13	1.359 (5)	C31—H31A	0.930
C12—H12A	0.930	C32—C33	1.466 (5)
C13—C14	1.415 (4)	C32—H32A	0.930
C13—H13A	0.930	C33—C34	1.386 (5)
C14—C19	1.425 (4)	C33—C38	1.389 (5)
C14—C15	1.433 (4)	C34—C35	1.376 (5)
C15—C16	1.369 (4)	C34—H34A	0.930
C16—O3	1.403 (4)	C35—C36	1.379 (6)
C16—C17	1.404 (4)	C35—H35A	0.930
C17—C18	1.348 (5)	C36—C37	1.370 (6)
C17—H17A	0.930	C36—H36A	0.930
C18—C19	1.420 (4)	C37—C38	1.383 (5)
C18—H18A	0.930	C37—H37A	0.930
C19—C20	1.409 (5)	C38—H38A	0.930
C20—H20A	0.930		
			
C10—C1—C2	120.2 (3)	C11—C20—H20A	119.6
C10—C1—H1A	119.9	C19—C20—H20A	119.6
C2—C1—H1A	119.9	O2—C21—O1	122.9 (3)
C3—C2—C1	121.2 (3)	O2—C21—C22	124.9 (3)
C3—C2—H2A	119.4	O1—C21—C22	112.2 (3)
C1—C2—H2A	119.4	C23—C22—C21	124.3 (3)
C2—C3—C4	120.7 (3)	C23—C22—H22A	117.9
C2—C3—H3A	119.6	C21—C22—H22A	117.9
C4—C3—H3A	119.6	C22—C23—C24	126.3 (3)
C9—C4—C3	118.1 (3)	C22—C23—H23A	116.8
C9—C4—C5	119.8 (2)	C24—C23—H23A	116.8
C3—C4—C5	122.1 (3)	C25—C24—C29	118.1 (3)
C6—C5—C4	117.9 (3)	C25—C24—C23	119.2 (3)
C6—C5—C15	121.2 (3)	C29—C24—C23	122.7 (3)
C4—C5—C15	120.8 (2)	C24—C25—C26	121.2 (4)
C5—C6—O1	119.7 (2)	C24—C25—H25A	119.4
C5—C6—C7	122.9 (3)	C26—C25—H25A	119.4
O1—C6—C7	117.2 (3)	C27—C26—C25	120.1 (4)
C8—C7—C6	119.8 (3)	C27—C26—H26A	120.0
C8—C7—H7A	120.1	C25—C26—H26A	120.0
C6—C7—H7A	120.1	C26—C27—C28	120.1 (3)
C7—C8—C9	120.7 (3)	C26—C27—H27A	119.9
C7—C8—H8A	119.6	C28—C27—H27A	119.9
C9—C8—H8A	119.6	C27—C28—C29	120.2 (4)
C10—C9—C4	119.6 (3)	C27—C28—H28A	119.9
C10—C9—C8	121.5 (3)	C29—C28—H28A	119.9
C4—C9—C8	118.8 (3)	C24—C29—C28	120.2 (4)
C1—C10—C9	120.2 (3)	C24—C29—H29A	119.9
C1—C10—H10A	119.9	C28—C29—H29A	119.9
C9—C10—H10A	119.9	O4—C30—O3	123.5 (3)
C20—C11—C12	119.7 (3)	O4—C30—C31	125.8 (3)
C20—C11—H11A	120.1	O3—C30—C31	110.7 (3)
C12—C11—H11A	120.1	C32—C31—C30	120.0 (3)
C13—C12—C11	121.5 (3)	C32—C31—H31A	120.0
C13—C12—H12A	119.2	C30—C31—H31A	120.0
C11—C12—H12A	119.2	C31—C32—C33	129.0 (3)
C12—C13—C14	120.6 (3)	C31—C32—H32A	115.5
C12—C13—H13A	119.7	C33—C32—H32A	115.5
C14—C13—H13A	119.7	C34—C33—C38	118.5 (3)
C13—C14—C19	117.6 (3)	C34—C33—C32	118.5 (3)
C13—C14—C15	123.0 (3)	C38—C33—C32	123.0 (3)
C19—C14—C15	119.4 (3)	C35—C34—C33	121.3 (4)
C16—C15—C14	118.2 (3)	C35—C34—H34A	119.3
C16—C15—C5	121.6 (2)	C33—C34—H34A	119.3
C14—C15—C5	120.2 (2)	C34—C35—C36	119.5 (4)
C15—C16—O3	117.3 (2)	C34—C35—H35A	120.3
C15—C16—C17	122.7 (3)	C36—C35—H35A	120.3
O3—C16—C17	119.9 (3)	C37—C36—C35	120.1 (3)
C18—C17—C16	119.4 (3)	C37—C36—H36A	119.9
C18—C17—H17A	120.3	C35—C36—H36A	119.9
C16—C17—H17A	120.3	C36—C37—C38	120.5 (4)
C17—C18—C19	121.5 (3)	C36—C37—H37A	119.7
C17—C18—H18A	119.2	C38—C37—H37A	119.7
C19—C18—H18A	119.2	C37—C38—C33	120.0 (3)
C20—C19—C18	121.8 (3)	C37—C38—H38A	120.0
C20—C19—C14	119.6 (3)	C33—C38—H38A	120.0
C18—C19—C14	118.5 (3)	C21—O1—C6	118.3 (2)
C11—C20—C19	120.9 (3)	C30—O3—C16	118.9 (2)
			
C10—C1—C2—C3	0.0 (5)	C17—C18—C19—C20	176.3 (3)
C1—C2—C3—C4	−0.6 (5)	C17—C18—C19—C14	−3.4 (5)
C2—C3—C4—C9	1.6 (4)	C13—C14—C19—C20	2.1 (4)
C2—C3—C4—C5	−175.3 (3)	C15—C14—C19—C20	−176.3 (3)
C9—C4—C5—C6	1.1 (4)	C13—C14—C19—C18	−178.2 (3)
C3—C4—C5—C6	177.9 (3)	C15—C14—C19—C18	3.4 (4)
C9—C4—C5—C15	−175.7 (2)	C12—C11—C20—C19	−1.1 (5)
C3—C4—C5—C15	1.1 (4)	C18—C19—C20—C11	179.6 (3)
C4—C5—C6—O1	−172.3 (2)	C14—C19—C20—C11	−0.7 (5)
C15—C5—C6—O1	4.4 (4)	O2—C21—C22—C23	165.2 (3)
C4—C5—C6—C7	1.7 (4)	O1—C21—C22—C23	−14.2 (4)
C15—C5—C6—C7	178.5 (3)	C21—C22—C23—C24	−175.0 (3)
C5—C6—C7—C8	−2.3 (4)	C22—C23—C24—C25	162.3 (4)
O1—C6—C7—C8	171.9 (3)	C22—C23—C24—C29	−15.3 (6)
C6—C7—C8—C9	0.0 (4)	C29—C24—C25—C26	3.2 (6)
C3—C4—C9—C10	−1.9 (4)	C23—C24—C25—C26	−174.4 (4)
C5—C4—C9—C10	175.0 (2)	C24—C25—C26—C27	−2.1 (7)
C3—C4—C9—C8	179.8 (3)	C25—C26—C27—C28	0.1 (7)
C5—C4—C9—C8	−3.3 (4)	C26—C27—C28—C29	0.7 (7)
C7—C8—C9—C10	−175.5 (3)	C25—C24—C29—C28	−2.4 (6)
C7—C8—C9—C4	2.7 (4)	C23—C24—C29—C28	175.1 (4)
C2—C1—C10—C9	−0.4 (5)	C27—C28—C29—C24	0.5 (7)
C4—C9—C10—C1	1.4 (4)	O4—C30—C31—C32	−4.8 (6)
C8—C9—C10—C1	179.6 (3)	O3—C30—C31—C32	174.8 (3)
C20—C11—C12—C13	1.3 (6)	C30—C31—C32—C33	179.8 (3)
C11—C12—C13—C14	0.2 (5)	C31—C32—C33—C34	165.9 (4)
C12—C13—C14—C19	−1.9 (4)	C31—C32—C33—C38	−15.7 (6)
C12—C13—C14—C15	176.5 (3)	C38—C33—C34—C35	0.0 (6)
C13—C14—C15—C16	−178.6 (3)	C32—C33—C34—C35	178.5 (3)
C19—C14—C15—C16	−0.3 (4)	C33—C34—C35—C36	0.9 (6)
C13—C14—C15—C5	1.1 (4)	C34—C35—C36—C37	−1.1 (6)
C19—C14—C15—C5	179.5 (2)	C35—C36—C37—C38	0.4 (6)
C6—C5—C15—C16	109.7 (3)	C36—C37—C38—C33	0.4 (6)
C4—C5—C15—C16	−73.6 (4)	C34—C33—C38—C37	−0.6 (5)
C6—C5—C15—C14	−70.1 (4)	C32—C33—C38—C37	−179.1 (3)
C4—C5—C15—C14	106.6 (3)	O2—C21—O1—C6	−15.5 (4)
C14—C15—C16—O3	−179.6 (2)	C22—C21—O1—C6	163.9 (2)
C5—C15—C16—O3	0.7 (4)	C5—C6—O1—C21	−69.4 (3)
C14—C15—C16—C17	−3.1 (4)	C7—C6—O1—C21	116.2 (3)
C5—C15—C16—C17	177.1 (3)	O4—C30—O3—C16	2.2 (5)
C15—C16—C17—C18	3.3 (5)	C31—C30—O3—C16	−177.5 (2)
O3—C16—C17—C18	179.6 (3)	C15—C16—O3—C30	−134.4 (3)
C16—C17—C18—C19	0.1 (5)	C17—C16—O3—C30	49.0 (4)

### Hydrogen-bond geometry (Å, °)

**Table:**

*D*—H···*A*	*D*—H	H···*A*	*D*···*A*	*D*—H···*A*
C23—H23A···O1	0.93	2.38	2.736 (3)	103
C32—H32A···O4	0.93	2.49	2.833 (4)	102
C11—C11A···Cg1^i^	0.93	2.85	3.746 (3)	162
C2—H2A···Cg2^ii^	0.93	2.74	3.507 (3)	140

Symmetry codes: (i) *x*+1, *y*, *z*; (ii) *x*−1/2, −*y*+3/2, −*z*.
